# Deuterated Linoleic Acid Attenuates the RBC Storage Lesion in a Mouse Model of Poor RBC Storage

**DOI:** 10.3389/fphys.2022.868578

**Published:** 2022-04-26

**Authors:** Christopher Y. Kim, Hannah Johnson, Sandy Peltier, Steven L. Spitalnik, Eldad A. Hod, Richard O. Francis, Krystalyn E. Hudson, Elizabeth F. Stone, Dominique E. Gordy, Xiaoyun Fu, James C. Zimring, Pascal Amireault, Paul W. Buehler, Robert B. Wilson, Angelo D’Alessandro, Mikhail S. Shchepinov, Tiffany Thomas

**Affiliations:** ^1^ Department of Pathology and Cell Biology, Columbia University Irving Medical Center, New York-Presbyterian Hospital, New York, NY, United States; ^2^ Bloodworks Research Institute, Seattle, WA, United States; ^3^ Institut National de la Transfusion Sanguine, Paris, France; ^4^ University of Virginia School of Medicine, Charlottesville, VA, United States; ^5^ X U1163, Laboratory of Cellular and Molecular Mechanisms of Hematological Disorders and Therapeutic Implications, INSERM, Université de Paris, Paris, France; ^6^ University of Maryland School of Medicine, Baltimore, MD, United States; ^7^ Department of Pathology and Laboratory Medicine, Perelman School of Medicine at the University of Pennsylvania, Philadelphia, PA, United States; ^8^ Department of Biochemistry and Molecular Genetics, University of Colorado School of Medicine, Aurora, CO, United States; ^9^ Retrotope Inc., Los Altos, CA, United States

**Keywords:** transfusion, RBC, deformability, ROS, lipidomics, peroxidation, oxylipins

## Abstract

**Background:** Long-chain polyunsaturated fatty acids (PUFAs) are important modulators of red blood cell (RBC) rheology. Dietary PUFAs are readily incorporated into the RBC membrane, improving RBC deformability, fluidity, and hydration. However, enriching the lipid membrane with PUFAs increases the potential for peroxidation in oxidative environments (e.g., refrigerated storage), resulting in membrane damage. Substitution of bis-allylic hydrogens with deuterium ions in PUFAs decreases hydrogen abstraction, thereby inhibiting peroxidation. If lipid peroxidation is a causal factor in the RBC storage lesion, incorporation of deuterated linoleic acid (DLA) into the RBC membrane should decrease lipid peroxidation, thereby improving RBC lifespan, deformability, filterability, and post-transfusion recovery (PTR) after cold storage.

**Study Design and Methods:** Mice associated with good (C57BL/6J) and poor (FVB) RBC storage quality received diets containing 11,11-D2-LA Ethyl Ester (1.0 g/100 g diet; deuterated linoleic acid) or non-deuterated LA Ethyl Ester (control) for 8 weeks. Deformability, filterability, lipidomics, and lipid peroxidation markers were evaluated in fresh and stored RBCs.

**Results:** DLA was incorporated into RBC membranes in both mouse strains. DLA diet decreased lipid peroxidation (malondialdehyde) by 25.4 and 31% percent in C57 mice and 12.9 and 79.9% in FVB mice before and after cold storage, respectively. In FVB, but not C57 mice, deformability filterability, and post-transfusion recovery were significantly improved.

**Discussion:** In a mouse model of poor RBC storage, with elevated reactive oxygen species production, DLA attenuated lipid peroxidation and significantly improved RBC storage quality.

## Introduction

Currently, the FDA allows human red blood cells (RBCs) to be stored at 1–6°C for up to 42 days in most preservation solutions (United States Food and Drug Administration, 1985). However, many biochemical and biomechanical changes during refrigerated storage decrease RBC quality. These include decreased adenosine triphosphate (ATP) and 2,3-diphosphoglycerate (2,3-DPG), and increased oxidative damage to membrane lipids and cytoskeletal and cytoplasmic proteins (Luten et al., 2008; Chaudhary and Katharia, 2012; Yoshida et al., 2019). Adverse biomechanical changes include reduced deformability, microparticle accumulation, increased aggregability, and transition from discocytes to echinocytes (Celle, 1969; Hovav et al., 1999; Berezina et al., 2002; Bosman et al., 2008). Collectively, these changes are termed the “storage lesion.”

The structural and biochemical changes associated with cold storage result in decreased RBC function and 24-h post-transfusion recovery (PTR) (Dumont and AuBuchon, 2008). During cold storage, the RBC membrane buds into microparticles, producing an irreversible loss of membrane surface area (Laczko et al., 1979). The loss of membrane surface area ultimately leads to the shape transition from discocyte to echinocyte to spherocyte, thereby reducing deformability and increasing adhesion to the endothelium (Burger et al., 2013; Roussel et al., 2017; Donadee et al., 2011; D'Alessandro et al., 2012). When transfused, these less deformable RBCs have difficulty traversing blood vessels due to decreased deformability and attenuated ATP release (Zhu et al., 2011). They are also more likely to be sequestered in the spleen due to their low deformability (Safeukui et al., 2018; Roussel et al., 2021). Additionally, they are more susceptible to splenic clearance by macrophage phagocytosis due to increased phosphatidylserine externalization and decreased expression of “don’t eat me” signals (e.g., CD47) (Oldenborg et al., 2001; Anniss and Sparrow, 2002; Burger et al., 2013). Functionally, the remaining circulating RBCs suffer from low concentrations of ATP and 2,3-DPG as well as morphological changes linked to aberrant membrane function in circulation. Collectively these critical changes in storage induced quality can contribute toward impaired perfusion and oxygenation at the tissue level (Apstein et al., 1985). Due to these deleterious effects, reducing the impact of the RBC storage lesion is important for improving clinical RBC transfusion outcomes.

The RBC membrane fatty acid composition can be directly modulated by altering dietary fatty acids. More specifically, increasing dietary long-chain polyunsaturated fatty acids (PUFAs), relative to saturated and monounsaturated fatty acids, will increase levels of the former in RBC membranes in a dose and time-dependent manner; indeed, changes are observed as early as 3 days after initiating high dose fish oil supplementation (Dougherty et al., 1987; Katan et al., 1997). Although benefits, such as increased membrane fluidity, can improve RBC deformability, PUFAs are particularly susceptible to the abstraction of bis-allylic hydrogens by reactive oxygen species (ROS). The resulting peroxyl radical can subsequently abstract hydrogens from neighboring PUFAs in the membrane, causing free radical lipid autoxidation (lipid peroxidation) (Barber and Bernheim, 1967). Increased oxidative stress occurs over time during RBC storage from the reaction of oxygen with ferrous iron; the storage lesion occurs when the rate of oxidative attacks exceeds the cellular antioxidant capacity. Although previous studies suggest that lipid peroxidation contributes to the storage lesion, the evidence has been indirect (Greenwalt et al., 1984; Kriebardis et al., 2008; Antonelou et al., 2010; Lippi and Sanchis-Gomar, 2019).

To demonstrate that lipid peroxidation contributes to the storage lesion, it is necessary to show an improved PTR when lipid peroxidation is attenuated during RBC storage. Previous studies have demonstrated that substitution with heavy hydrogen (deuterium) at the bis-allylic position(s) of PUFAs provides protection against hydrogen abstraction (Hill et al., 2012; Beaudoin-Chabot et al., 2019; Firsov et al., 2019). Collectively, in these studies, bis-allylic deuterium substitution in PUFAs significantly decreased lipid peroxidation, whereas substitution at the mono-allylic site(s) provided no protection (Hill et al., 2011; Hill et al., 2012; Shchepinov et al., 2014; Beaudoin-Chabot et al., 2019; Firsov et al., 2019). To assess whether lipid peroxidation is causal for the RBC storage lesion, the current study investigated whether feeding mice a diet highly enriched in 11,11-d2 linoleic acid (DLA), as compared to a control diet, protects against lipid peroxidation *in vivo*, as well as during refrigerated storage. To this end, dietary experiments were performed using mice exhibiting good (C57BL/6J) and poor (FVB) RBC storage quality; outcome variables evaluated included RBC lifespan *in vivo*, 24-h PTR before and after storage, RBC deformability, and RBC lipid peroxidation.

## Materials and Methods

### Chemicals and Standards

Deuterated and control diets were obtained from Research Diets, Inc (D20100101 and D20100202, New Brunswick, NJ). 11,11-d2 linoleic acid ethyl ester (DLA-EE) was provided by Retrotrope Inc. EZ-Link™ Sulfo-NHS-LC-Biotin (21,335, biotinylating reagent) and APC streptavidin were purchased from Thermo Fisher Scientific (349,024, Waltham, MA), and BD Biosciences (Franklin Lakes, NJ), respectively. Phosphate buffered saline, 1X (PBS; 21-040-CM was purchased from Corning (Corning, NY). Leukoreduction filters (AP-4952) were purchased from Pall (Port Washington, NY).

Fatty acid methyl esters (FAME) standard mixture (FAME 25) was purchased from USP (1269119; Rockville, MD), while methyl esters of arachidonic acid (AA, 2566-89-4), docosahexaenoic acid (DHA, 2566-90-7), docosapentaenoic acid (DPA, 108698-02-8), eicosapentaenoic acid (EPA, 2734-47-6), and nonadecanoate (C19:0, 1731-94-8), as well as malondialdehyde (MDA, 100,683-54-3), were purchased from Sigma (St. Louis, MO). Malondialdehyde-D2 (MDA-D2, D-6469) was purchased from CDN Isotopes (Pointe-Claire, QC, Canada). Heptane (34,873), hexane (34,859), pentane (34,956), methanol (34,860), and hydrochloric acid in methanol (90,964) were purchased from Sigma. Liquid chromatography-mass spectrometry (LC-MS) grade water was purchased from Supelco (Bellefonte, PA, WX0001). Chloroform was purchased from J.T. Baker (67-66-3, Phillipsburg, NJ).

### Animal Experiments

All mouse experiments were approved by the International Animal Care and Use Committee (IACUC) at Columbia University Irving Medical Center (AABH6563). Two different mouse strains (C57BL/6J, Jackson Laboratory, and FVB, Charles Rivers) were fed diets *ad libitum*, containing linoleic acid ethyl ester (LA-EE; control) or DLA-EE (experimental). After a 2-week acclimation period on a regular chow diet, 10-week-old female mice were fed control or experimental diets for 8 weeks (*n* = 15 mice/group). The AIN93M modified diets are casein-based with 13, 22, and 65% of the calories coming from protein, fat, and carbohydrate, respectively. The fat sources include coconut oil (7 g/100 g), sunflower oil (2 g/100 g), flax seed oil (0.5 g/100 g) and only differ in the inclusion of deuterated or non-deuterated linoleic acid ethyl ester (1 g/100 g). Mice were on a 12:12 light/dark cycle. Mouse body and food weights were recorded two times per week. Female C57BL/6J mice ubiquitously expressing GFP (C57BL/6J-TG UBC-GFP) were acquired from Jackson Labs for loading controls in the PTR experiments. No sex differences in response to DLA treatment were anticipated. Although RBCs from female humans and mice have been shown to enhance post-transfusion recovery (PTR), female mice were arbitrarily chosen for use as blood donors as well as recipients.

### *In vivo* RBC Lifespan

At the start of the fifth week of feeding, RBCs were biotinylated *in vivo* (1 mg/300 μl in PBS injected into the retro-orbital sinus) (Dholakia et al., 2015). Blood (1 ul) was collected from the tail vein after 1, 3, 7, 17, and 21 days and labeled with APC streptavidin (1:100 v/v in PBS) for 40 min at room temperature. Samples were washed and resuspended in 1 ml of PBS and evaluated by flow cytometry (see below). RBC lifespan was determined by plotting the number of biotin-positive RBCs relative to the number of total RBCs. These flow data from days 1–21 were analyzed by simple linear regression with the regression line extrapolated to the *x*-intercept to estimate the *in vivo* RBC lifespan for each diet group; the slope of the line represented the fractional RBC removal rate.

### Blood Collection and Storage

On the day before blood collection, all RBCs were re-biotinylated *in vivo*, as above. After 8 weeks of feeding with control or experimental diets, blood was collected aseptically from mice *via* cardiac puncture, pooled by diet and mouse strain, and CPDA-1 was added (14% by volume). Leukocytes were removed *via* filtration using passive gravitational flow, the resultant blood was packed to 60% hematocrit and then blood was stored for 6 (for FVB) or 12 days (for C57BL/6J) at 4°C in 5.0 ml microcentrifuge tubes. Blood storage times were selected based on previous data that most closely predicted the FDA minimum requirement for 24-h PTR for each mouse strain (i.e., >75%) (Zimring et al., 2014). Blood units were prepared at Columbia University Irving Medical Center in New York City, NY. Fresh aliquots of the blood units (100 ul) were sent by international shipping (2-day transport) to Paris, France for deformability (LORRCA) and filterability (microsphiltration) experiments. Temperature (4°C) was maintained during transportation using cold packs.

###  24-Hr PTR

On the morning of the PTR experiments, a blood unit was prepared from UBC-GFP mice as above with a final hematocrit of 60%. Fresh (1-day stored) and stored blood units were spiked 1:5 with fresh UBC-GFP blood units as a loading control. The final hematocrit of these units was 60%. Aliquots (200 μl) from these spiked blood units were transfused *via* the retro-orbital venous sinus into 10-week-old female mice consuming a chow diet (*n* = 10/group). Blood samples were collected *via* tail puncture (1 μl in 1 ml PBS) at 5 min, 120 min, and 24 h after transfusion and processed using the same workflow described for analyzing *in vivo* RBC lifespan. PTR was measured using flow cytometry (see below).

### Flow Cytometry

Flow cytometry was conducted using an Attune NxT with the No-Wash No-Lyse Filter kit (ThermoFisher Scientific; United States) to detect RBCs. Aliquots (200 μl) of RBCs (1 μl/1 ml PBS) were acquired at a flow rate of 100 μl/min; 100,000 events were collected. The same acquisition settings were used for measuring both RBC lifespan and PTR.

The *in vivo* RBC lifespan was determined as the percentage of biotin-positive RBCs in the total RBC count.

PTR was measured as the ratio of biotin-positive RBCs to GFP-positive RBCs in circulation 24 h-post transfusion relative to the same ratio in the spiked blood unit.

### Analysis of Fatty Acid Methyl Esters (FAMEs) by Gas Chromatography/Mass Spectrometry (GS-MS)

A frozen aliquot (−80°C) of RBCs (20 μl) were allowed to thaw to room temperature and lipids were extracted using a modified Folche method. For diet analysis, two pellets of each diet (approximately 3 g) were pulverized with a mortar and pestle. In a 1.5 ml microcentrifuge tube, 600 μl of 2:1 chloroform/methanol, 100 μl of liquid chromatography/mass spectrophotometry (LC-MS) grade water, and 20 μl of C19:0 methyl ester were added to 20 ul of RBCs or 50 mg of diet, vortexed for 10 min (room temperature), then centrifuged at 14,000 x g for 10 min (4°C). The supernatant and protein disc were discarded while the bottom lipid layer was transferred to a 13X100 Pyrex screw cap tube. The lipids were evaporated to dryness under nitrogen gas. The dried samples were transesterified to fatty acid methyl esters (FAMEs) with 100 μl of methanolic-HCL (100°C for 1 h).

The FAMEs were extracted with hexane/water (2:1). The organic layer was transferred to a new tube and dried with sodium sulfate (∼1 g). The dried solution was decanted into a new tube and dried under nitrogen gas. The sample was solubilized in 500 μl of heptane and transferred to a 2 ml GC vial.

FAMEs were separated by a DB-FATWAX UI column (30m, 250 μM diameter, 0.25 μM film thickness, Agilent Technologies, G3903-63008) using a 5975c GC/MS (Agilent Technologies, Santa Clara, CA). 1 ul of the sample was injected (pulsed splitless) into the GC with the following settings: inlet temp 250°C; flow rate 1.0 ml/min; transfer line 280°C; oven program mode was 80°C, held for 1.5 min, then ramped to 240°C (3°C/min) and held for 7 min. Quantification of FAMES was done using selected ion monitoring (SIM) mode of m/z 55, 67, 69, 74, and 79. Quantification was done using the GC-MS Agilent software.

### Analysis of Malondialdehyde (MDA) by GC-MS

A frozen aliquot (−80°C) of each of the blood banks (25 μl) was allowed to thaw to room temperature. 25 μl of malondialdehyde-d2 (MDA-d2,10 μg/ml in methanol), 100 μl of 2,4-dinitrophenylhydrazine (DNPH, 15.7M in 2M HCl, Sigma, D199303), and 200 μl of LC-MS grade water was added to the blood bank and mixed for 15 min 2ml of pentane were added and the solution was mixed for 15 min. The organic layer was transferred to a new vial and dried with anhydrous sodium sulfate, decanted to a new tube, evaporated to dryness with nitrogen (60°C). The sample was resolubilized in 100 μl of chloroform and transferred to a fixed-insert GC vial.

MDA was separated using an HP-5MS column (25m, 200 μM diameter, 0.5 M film thickness, Agilent, 19091S-433) using a 5975c GC/MS (Agilent Technologies, Santa Clara, CA). 1 ul of the sample was injected (pulsed splitless) into the GC with the following settings: inlet temp 250°C; flow rate 1.0 ml/min; transfer line 280°C; oven program mode was 80°C, held for 1 min, then ramped to 280°C (7.5°C/min) and subsequently ramped to 280°C (20°C/min) after which it was held for 3 min. The injection volume was 1 μl in pulsed splitless mode. Quantification of MDA was done using selected ion monitoring (SIM) mode of m/z 158, 160, 204, 206, 234, and 236. Quantification was done using the GC-MS Agilent software.

### RBC Deformability

RBC elongation index (EI) was measured by ektacytometry using a laser-assisted optical rotational red cell analyzer (LORRCA, RR Mechatronics, Netherlands) to measure the diffraction pattern of sheared RBC (over a shear stress range of 0.3–30Pa) as described previously (Bessis et al., 1980). EI was defined as the ratio of the difference between the two axes of the ellipsoidal diffraction pattern and the sum of these two axes. Control samples were fresh C57BL/6 RBCs. Deformability was performed on fresh (2 days stored) and stored (6 or 12 days stored for FVB or C57 mice, respectively).

### Microsphiltration

Microsphiltration plates were prepared as described previously (Marin et al., 2021). Calibrated metal microspheres 5–25 μm in diameter that form a matrix that mimics flow through interendothelial slits of the spleen was used to assesses the deformability of mixtures of 5% unstained “test” RBCs and 95% CFSE-stained fresh “diluent” RBCs in a Krebs-albumin solution (Krebs-Henseleit buffer, Sigma-Aldrich) modified with 2 g of glucose, 2.1 g of sodium bicarbonate, 0.175 g of calcium chloride dehydrate, and 5 g of lipid-rich bovine serum albumin (Albu-MAX II, Thermo Fisher Scientific) for 1 L of sterile water (pH 7.4).

RBC suspensions were then filtered through the microsphere layer. The upstream and downstream proportion of unstained test RBCs was evaluated by flow cytometry (Canto II, BD Biosciences) and analyzed with computer software (FlowJo, V10, BD Biosciences). The retention rate was calculated using the formula:

Δ = {[(% of test RBCs in upstream sample)—(% of test RBCs in downstream sample)]/(% of test RBC in the upstream sample)}× 100.

Positive values, named retention, indicate that sampled RBCs are less deformable than diluent RBCs, whereas negative values, named enrichment, indicate that sampled RBCs are more deformable compared to diluent RBCs. To validate each experiment, control samples were fresh C57BL/6 RBCs (negative control) and 0.8% glutaraldehyde-fixed RBCs (positive control). Microsphiltration was performed on fresh (2 days stored) and stored (6 or 12 days stored for FVB or C57 mice, respectively).

### Analysis of Free Polyunsaturated Fatty Acids (PUFAs), Oxylipins, Lysophospholipids, and 4-Hydroxy-Nonenal-Glutathione (4-HNE-GSH) by Liquid Chromatography-Tandem Mass Spectrometry

Free PUFAs, oxylipins, and lysophospholipids in fresh and stored RBCs were analyzed by liquid chromatography-tandem mass spectrometry (LC-MS/MS) as described previously (Fu et al., 2016; Fu et al., 2019; Medved et al., 2021). Briefly, analytes were extracted by 80% methanol (vol/vol) with an internal standard mixture containing arachidonic acid-d8 (AA-d8), docosahexaenoic acid-d5 (DHA-d5), eicosapentaenoic acid-d5 (EPA-d5), dihomo-γ-linolenic acid-d6 (DGLA-d6), α-linolenic acid-d14 (ALA-d14), linoleic acid-d4 (LA-d4), oleic acid-d17 (OA-d17), 15-hydroxyeicosatetraenoic acid-d8 (15-HETE-d8), 5-hydroxyeicosatetraenoic acid-d8 (5-HETE-d8), 12-hydroxyeicosatetraenoic acid-d8 (12-HETE-d8), 9-hydroxyoctadecadienoic acid-d4 (9-HODE-d4), 13-hydroxyoctadecadienoic acid-d4 (13-HODE-d4), 9,10-dihydroxyoctadecenoic acid-d4 (9,10-diHOME-d4), 12,13-dihydroxyoctadecenoic acid-d4 (12,13-diHOME-d4) from Cayman Chemical (Ann Arbor, MI); 17:1 lysophosphatidylcholine (17:1 LPC), 17:1 lysophosphatidylethanolamine (17:1 LPE), 17:1 lysophosphatidylserine (17:1 LPS), and 17:1 lysophosphatidylinositol (17:1 LPI) from Avanti Polar Lipids (Alabaster, AL). Liquid chromatography-tandem mass spectrometry (LC-MS/MS) analysis was performed using a mass spectrometer (QTrap 6500, AB Sciex, Framingham, MA) coupled with an ultra-performance liquid chromatographer (Acquity I-Class, Waters, Milford, MA). Analytes were separated on a C18 column (Acquity HSS T3, 2.1 × 100 mm, 1.8 μm, Waters). The mobile phase was composed of (A) water/acetonitrile (95/5, vol/vol) with 5 mmol/L ammonium acetate and (B) 2-propanol/acetonitrile/water (50/45/5, vol/vol/vol) with 5 mmol/L ammonium acetate. Analytes were eluted at a flow rate of 0.3 ml/min using the following gradient: 0–2 min, 20–40% B; 2–12 min, 40–80% B; 12–14 min, 80–98% B; 14–17 min, 98% B. The column temperature was maintained at 40°C and the autosampler temperature at 8°C. Analytes were detected using multiple reaction monitoring (MRM) in the negative ion mode and were quantified relative to their deuterium-labeled or 17:1 lysophospholipids analogs. For oxylipins without a deuterium-labeled analog, 15-HETE-d8 was used for quantification.

The lipid peroxidation product 4-hydroxynonenal-glutathione (4-HNE-GSH) was analyzed by LC-MS/MS. Analytes were extracted the same as above. The mobile phase was composed of (A) 0.1% formic acid in water and (B) 0.1% formic acid in acetonitrile. Analytes were eluted at a flow rate of 0.3 ml/min using the following gradient: 0–2 min, 2–5% B; 2–12 min, 5–95% B; 12–14 min, 95–99% B. 4-HNE-GSH was detected using MRM in the positive ion mode using m/z 464.2 for the precursor ion and m/z 308.1 for the product ion. The concentration was quantified using peak area by 4-HNE-GSH external standard calibration (Cayman Chemical). Data were collected and processed as above.

Data were collected and processed using computer software (Analyst Version 1.6.2, and MultiQuant Version 2.1.1, AB Sciex).

### Statistics

Statistical analysis was performed using Prism 9 (GraphPad; San Diego, CA). Statistical tests used for each experiment are defined in the figure legends. Flow cytometry data were analyzed using FlowJo 10 (BD Biosciences; Ashland, OR).

## Results

### Dietary Lipids Are Incorporated Into the RBC Membrane in C57BL/6J and FVB Mice

**FIGURE 1:**
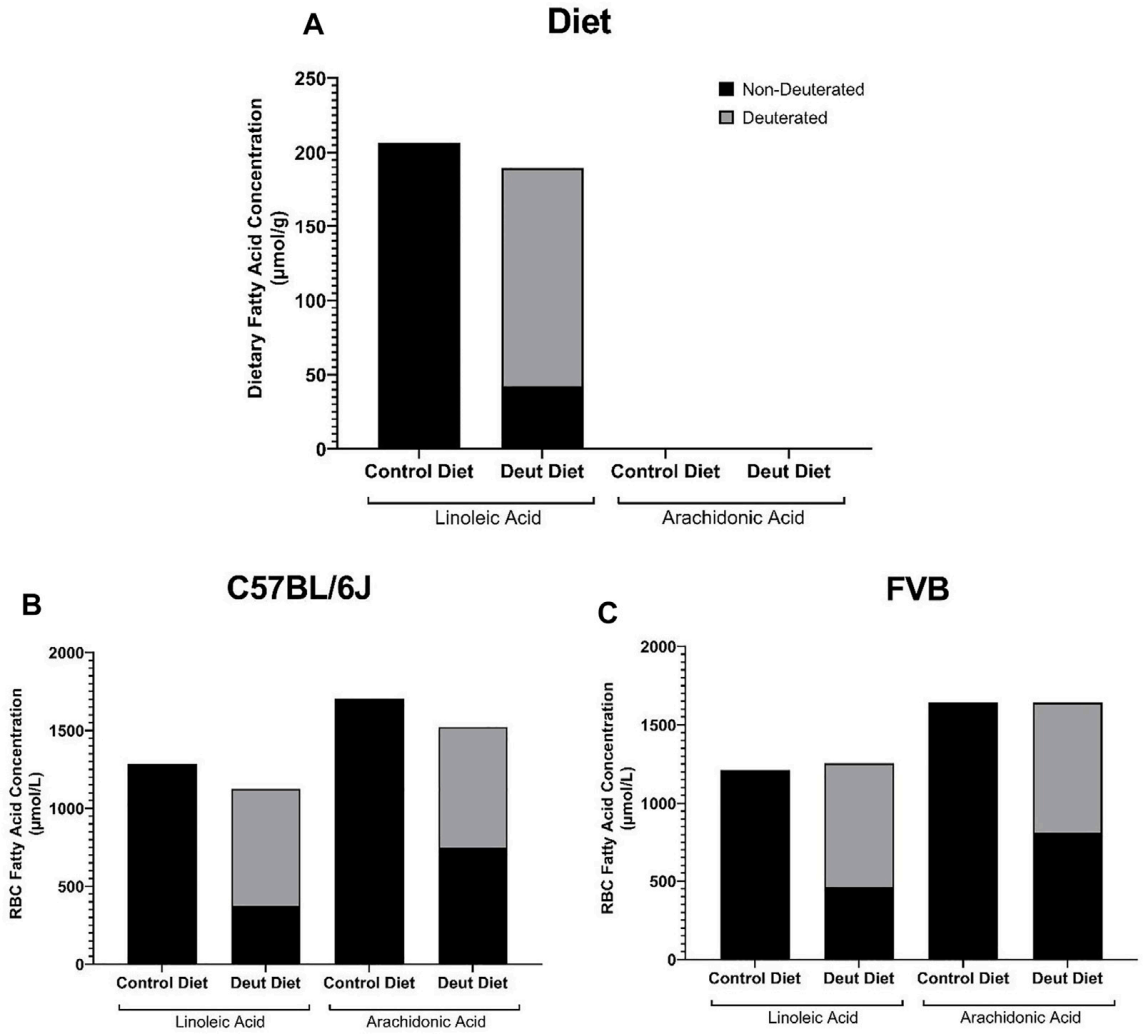
Deuterated PUFAs in diets and mouse RBCs. The contributions of DLA and AA to the experimental diets **(A)**, as well as to the RBCs from C57BL/6J **(B)** and FVB mice **(C)** prior to storage, were evaluated by GC-MS and LC-MS/MS.

First, we sought to investigate the RBC fatty acid profiles of C57BL/6J and FVB mice fed AIN-93M modified diets that differed only in deuterated vs. non-deuterated-LA-EE. They were equally palatable to C57BL/6J and FVB mice with no differences in food consumption (Supplementary Figures 1A, 2A) or body weight (Supplementary Figures 1B, 2B). Rodent diets are highly enriched in saturated and monounsaturated medium and long-chain fatty acids with no appreciable contribution of very long-chain PUFAs (AA (20:4 n-6), EPA (20:5 n-3), DPA (22:5 n-3), and DHA (22:6 n-3); Table 1). No differences in the concentrations of any of the fatty acids, including LA (18:2 n-6) and alpha-linolenic acid (LNA; 18:3 n-3) were observed in the control compared to the deuterated diet. Mass spectrometry makes it straightforward to distinguish deuterated and non-deuterated forms of compounds due to the differences in their respective mass-to-charge ratios. Since LA-EE was not the sole source of LA in the diets, only 77.8% of LA was in the deuterated form in the DLA diet (Figure 1A). No DLA was detected in the control diet and no other deuterated forms of other fatty acids, including AA, were detected in either of the diets (Figure 1A).

**TABLE 1:** Fatty acid profiles of rodent diets and of the fresh (unstored) RBCs obtained from FVB and C57BL/6J mice that consumed control or deuterated linoleic acid diets for 8 weeks.

				C57		FVB	
		Diet		Fresh RBC			
		Control Diet	Deut Diet	Control Diet	Deut Diet	Control Diet	Deut Diet
Total Fatty Acid		*(umol/g)*		*(umol/L)*			
C12	Lauric Acid	349.8	253.4	<0.1	<0.1	<0.1	<0.1
C14	Myristic Acid	73.8	47.6	151.6	184.0	122.1	151.1
C16	Palmitic Acid	40.9	30.6	4,543.4	4,131.1	4,208.0	5,584.7
016:1	Palmitoleic Acid	28.9	21.8	101.7	90.4	79.4	131.9
C18	Stearic Acid	46.6	49.4	1,482.1	1,180.1	1,234.5	1,294.5
C18:1	Oleic Acid	93.5	91.6	1,644.1	1,598.8	1,742.1	1,613.5
C18:2	Linoleic Acid (LA)	206.4	189.5	1,284.4	1,124.9	1,116.9	1,132.6
C18:3	Linolenic Acid (LNA)	1.3	1.3	11.4	5.1	11.2	11.7
C20:1	Eicosenoic Acid	0.4	0.5	39.3	30.6	38.9	42.8
C20:2	Eicosadienoic Acid	0.1	0.1	17.7	15.9	17.7	17.4
C20:3	Dihomo-y-linolenic acid (DGLA)	0.1	0.1	160.9	149.0	161.8	174.1
020:4	Arachidonic Acid (AA)	0.1	0.2	1,703.4	1,522.8	1,593.0	1,637.6
C20:5	Eicosapentaenoic Acid (EPA)	0.1	0.0	71.8	127.7	79.2	79.5
C22:5	Docosapentaenoic Acid (DPA)	0.1	0.0	71.5	69.6	76.2	92.3
C22:6	Docosahexaenoic Acid (DHA)	0.1	0.1	667.4	571.6	548.6	627.4

Although lauric acid (C12:0) was the most abundant fatty acid in the diet, it was not detected in the RBC samples (Table 1). Dietary LA and LNA were elongated and desaturated *in vivo* into n-6 (AA) and n-3 PUFAs (EPA, DPA, DHA) and incorporated into the RBC membrane in both C57BL/6J and FVB mice (Table 1). No differences were observed in RBC fatty acid profiles as a function of DLA treatment or strain differences (Table 1). Total RBC LA and AA did not differ between control and DLA diet groups in either strain (Table 1; Figures 1B,C). The percentage of DLA in RBCs was 66.7 and 63% and deuterated AA (13,13-D2-AA) was 50.8 and 50.5% for C57BL/6J and FVB mice consuming DLA diets, respectively (Figures 1B,C). No deuterated LA or AA was observed in RBCs from control mice of either strain. Together, these results demonstrate that DLA fed to C57BL/6J and FVB mice were incorporated into RBC membranes.

### The DLA Diet was Not Associated With Improved RBC Lifespan

**FIGURE 2:**
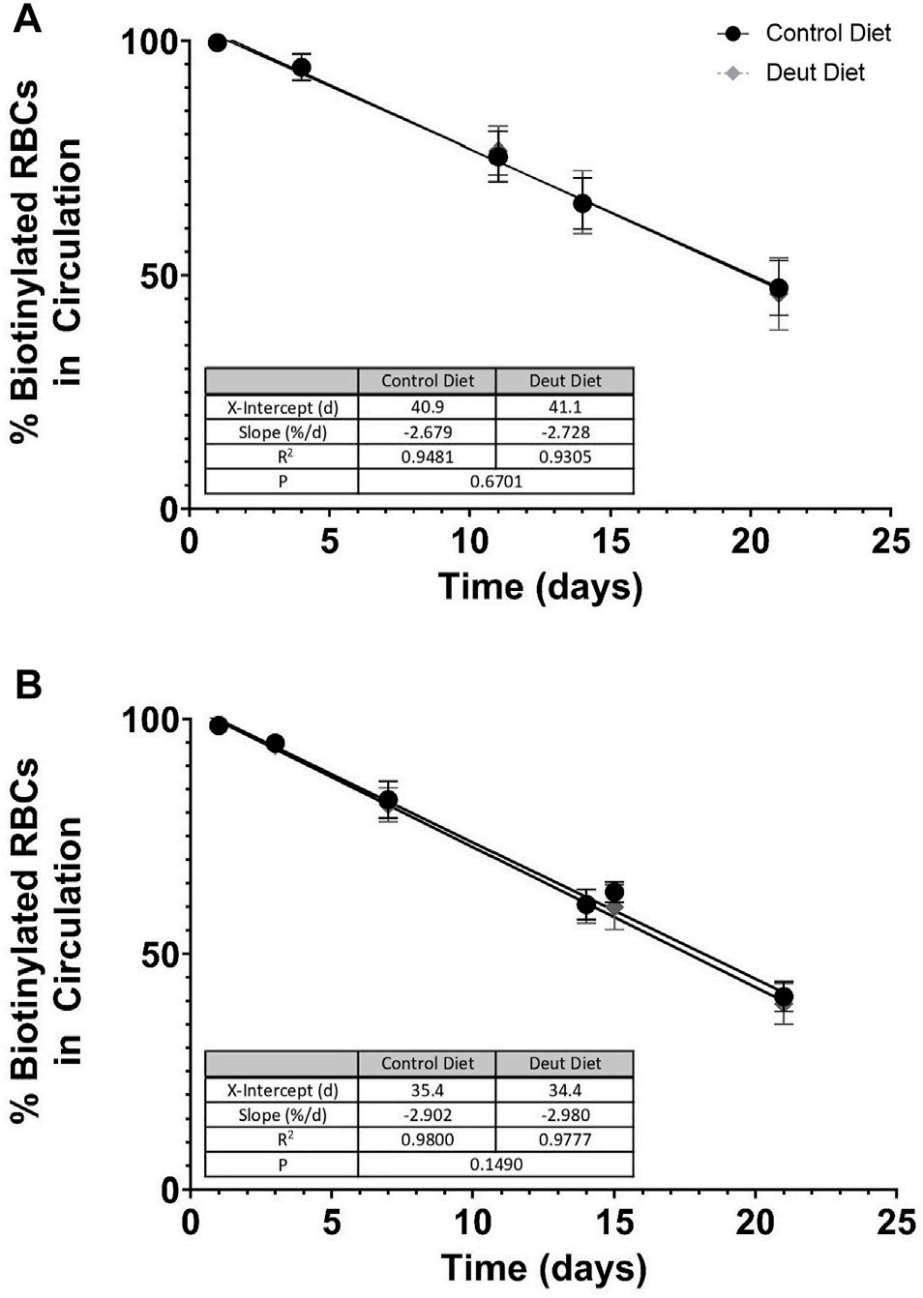
RBC lifespan *in vivo*. RBCs were biotinylated *in vivo* after consuming experimental diets for 5 weeks. Serial blood samples were taken *via* tail puncture on days 1, 3, 7, 14, and 21. At each time point, the total percentages of biotinylated RBCs circulating in C57BL/6J mice **(A)** and FVB mice **(B)** were quantified by flow cytometry (*n* = 15/group). Flow data from days 1–21 were analyzed by simple linear regression with the regression line extrapolated to the *x*-intercept to estimate the *in vivo* RBC lifespan for each diet group, with the slope of the line representing the fractional RBC removal rate.

We next examined the lifespan of RBCs from mice fed the DLA diet compared to the control diet. RBCs were biotinylated *in vivo*, and the resulting clearance of labeled cells was evaluated over 3 weeks. The slope (fractional RBC removal rate) and *x*-intercept (inferred linear RBC lifespan) of RBC survival data did not significantly differ between control and DLA diet groups in either C57BL/6J (control diet: 2.679% RBCs cleared/day, inferred lifespan of 40.9 days; DLA diet: 2.728% RBCs cleared/day, inferred lifespan of 41.1 days) or FVB (control diet: 2.902% RBCs cleared/day, inferred lifespan of 35.4 days; DLA diet: 2.980% RBCs cleared/day, inferred lifespan of 34.4 days) mice. However, the slope was flatter and *x*-intercept larger in C57BL/6J (Figure 2A) as compared to FVB (Figure 2B) groups.

### Lipid Peroxidation was Decreased in Mice Consuming DLA Diets

**FIGURE 3:**
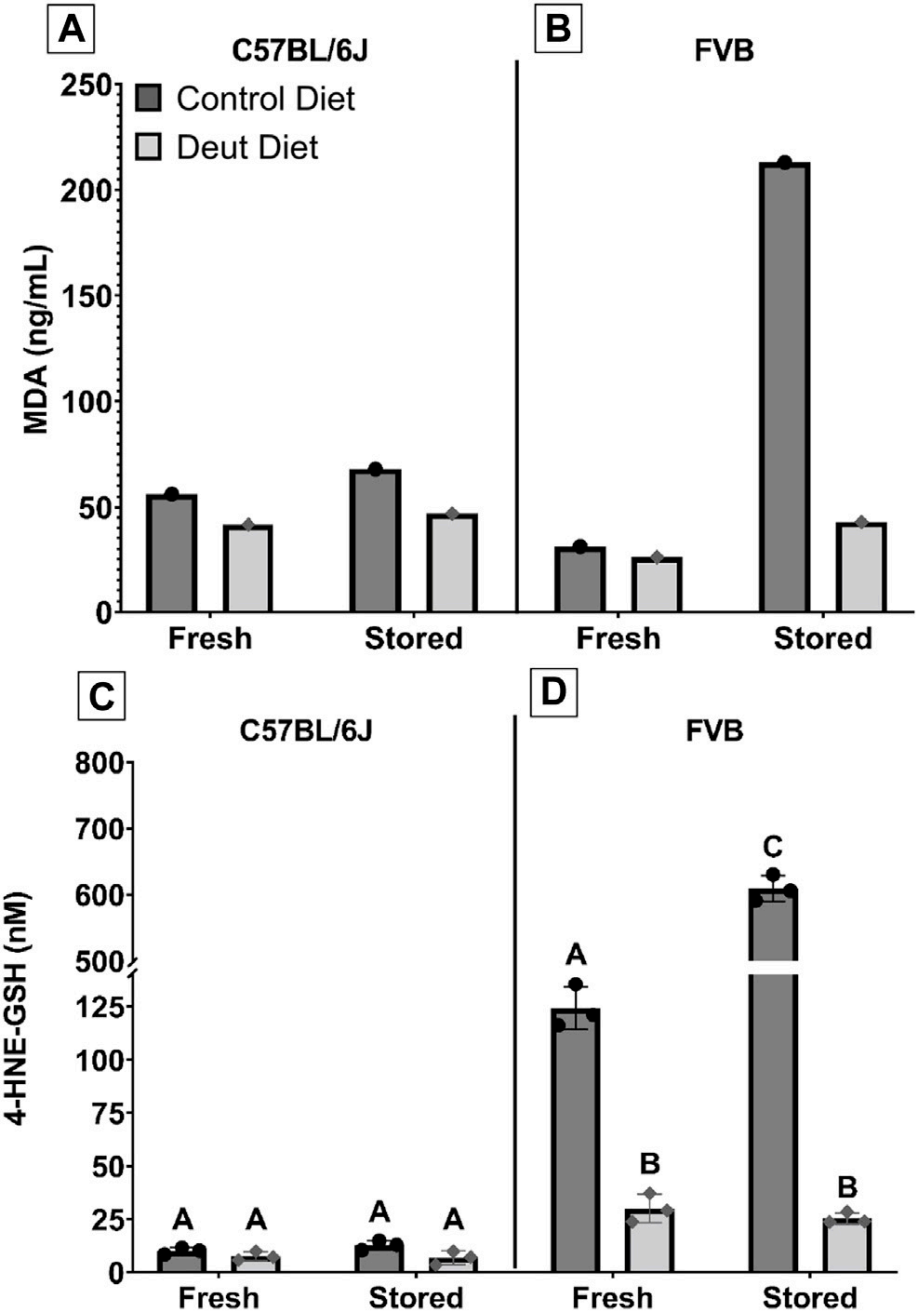
MDA and 4-HNE-GSH in fresh and stored RBCs. MDA, an end-product of lipid peroxidation, was extracted from both fresh and stored blood samples from C57BL/6J **(A)** and FVB mice **(B)**, and quantified by GC-MS. Another lipid peroxidation marker, 4-HNE-GSH, was extracted from fresh and stored blood banks prepared from C57BL/6J **(C)** and FVB **(D)** mice and quantified by LC-MS/MS. The scaling for 4-HNE-GSH was altered to allow for graphical visualization of the data for groups with levels greater than 150 nM. Groups that do not share common letters are statistically different from one another by one-way ANOVA with Tukey’s multiple comparisons follow-up test (*p* < 0.05).

We next investigated whether RBC lipid peroxidation was affected in mice fed the DLA diet compared to the control diet. MDA, an end product of lipid peroxidation of PUFAs, was evaluated in fresh and stored blood units. In C57BL/6J RBCs, MDA increased by 21% in the control blood unit after 12 days of storage but only 12% in the DLA group. Compared to the control group, MDA was 25 and 30% lower in the DLA group in fresh and 12-day stored blood, respectively in C57BL/6J RBCs (Figure 3A). In FVB RBCs, MDA increased by 590% in the control group after 6 days of storage as compared to only 59% in the DLA group. DLA was associated with a modest 12% reduction of MDA in the fresh FVB blood unit but an 80% reduction after 6 days of storage (Figure 3B).

4-hydroxynonenal (4-HNE) is another end product of lipid peroxidation; however, it is not stable and has a short half-life. The 4-HNE-glutathione adduct (4-HNE-GSH) has a longer half-life and is more amenable to quantification by LC-MS/MS. (D’Alessandro et al., 2021). Similar to our MDA observations, DLA supplementation was associated with a relatively small decrease in 4-HNE-GSH before (24%) and after 12-day of storage (46%; Figure 3C) in C57BL/6J RBCs but a striking decrease in FVB RBCs for fresh (76%) and 6-day stored blood (96%; Figure 3D).

**FIGURE 4:**
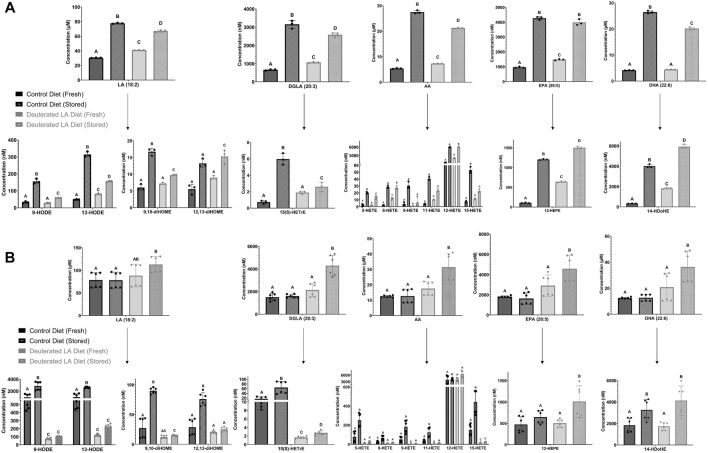
Free PUFAs in fresh and stored RBCs. Free PUFAs, along with their respective bioactive metabolites (i.e., oxylipins), were measured in fresh and stored blood banks prepared from both C57BL/6J **(A)** and FVB mice **(B)**. The experiment was repeated twice for FVB mice (2 biological replicates) and each blood bank was analyzed 3 times (3 technical replicates/biological replicate). Breaks in scaling were applied to some of the oxylipin species to allow for graphical visualization of the data for all groups. Groups that do not share common letters are statistically different from one another by one-way ANOVA with Tukey’s multiple comparisons follow-up test (*p* < 0.05).

Oxylipins are formed both enzymatically and non-enzymatically from RBC PUFAs. The non-enzymatically derived oxylipins formed from LA, dihomo-γ-linolenic acid (DGLA), and AA followed the same trend as was observed for MDA and 4-HNE-GSH (Figure 4). In both C57BL/6J and FVB mice, levels of hydroxyeicosatetraenoic acids (HETEs) and hydroxy-octadecadienoic acids (HODEs) were all higher in fresh control blood as compared to DLA blood. Following storage, DLA was associated with a smaller increase in several non-enzymatically derived oxylipins compared to controls, with the largest attenuation observed in FVB mice (e.g., 13-HODE, 9,10-dihydroxy-octadecenoic acid (diHOME), and 11-HETE).

### Lysophospholipids and Free Fatty Acids Increase After Storage

**FIGURE 5:**
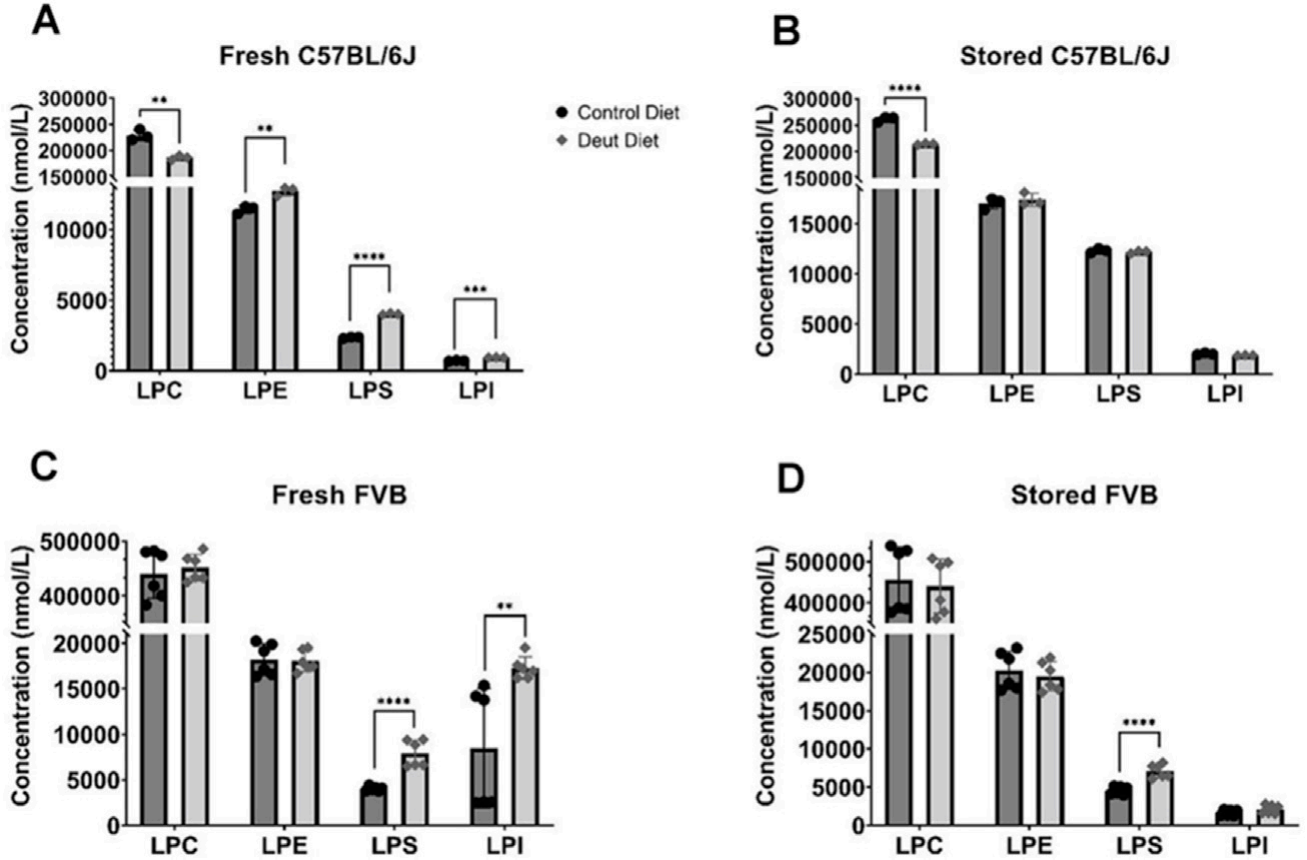
Lysophospholipids. Lysophospholipids were quantified in fresh and stored RBCs using LC-MS/MS. Data for C57BL/6J mice **(A, B)** reflect one biological replicate (3 technical replicates) and for FVB mice **(C, D)** reflect two biological replicates (3 technical replicates/experiment). Each blood bank is from 15 donor mice. Group differences were analyzed by unpaired T-Test (**p < 0.01; ***p < 0.001; ****p < 0.0001).

We then explored whether feeding mice DLA diets affects lysophospholipids and free fatty acids in stored RBC blood units. No differences in total RBC fatty acids were observed in either strain using either diet in fresh blood (Table 1). Before storage, all lysophospholipid (LPL) species (lysophosphatidylcholine (LPC), lysophosphatidylethanolamine (LPE), lysophosphatidylserine (LPS), and lysophosphatidylinositol (LPI)) were elevated in RBCs from DLA C57BL/6J mice relative to control mice (Figure 5A). After 12 days of storage, the levels of all LPL species increased in both control and DLA groups relative to fresh levels. LPS and LPI increased more in the control group compared to the DLA group such that only LPC remained different after storage (Figure 5B). In RBCs from FVB mice, LPS and LPI were higher in the DLA compared to the control group prior to storage (Figure 5C). After 6 days of storage, only LPS remained significantly higher in the DLA group (Figure 5D).

Consistent with elevated levels of LPLs in RBCs from C57BL/6J mice consuming a DLA diet, all levels of free PUFAs, except DHA, were elevated in the DLA group (Figure 4). After storage, PUFA levels increased in both control and DLA groups in C57BL/6J mice; however, the increase was higher in the control group relative to the DLA group (Figure 4A). In FVB mice, there were no differences in free PUFAs. After storage, free PUFAs were higher in the DLA group, but not in the control group (Figure 4B).

### DLA is Associated With Improved Deformability and Filterability in FVB but Not C57 Mice

**FIGURE 6:**
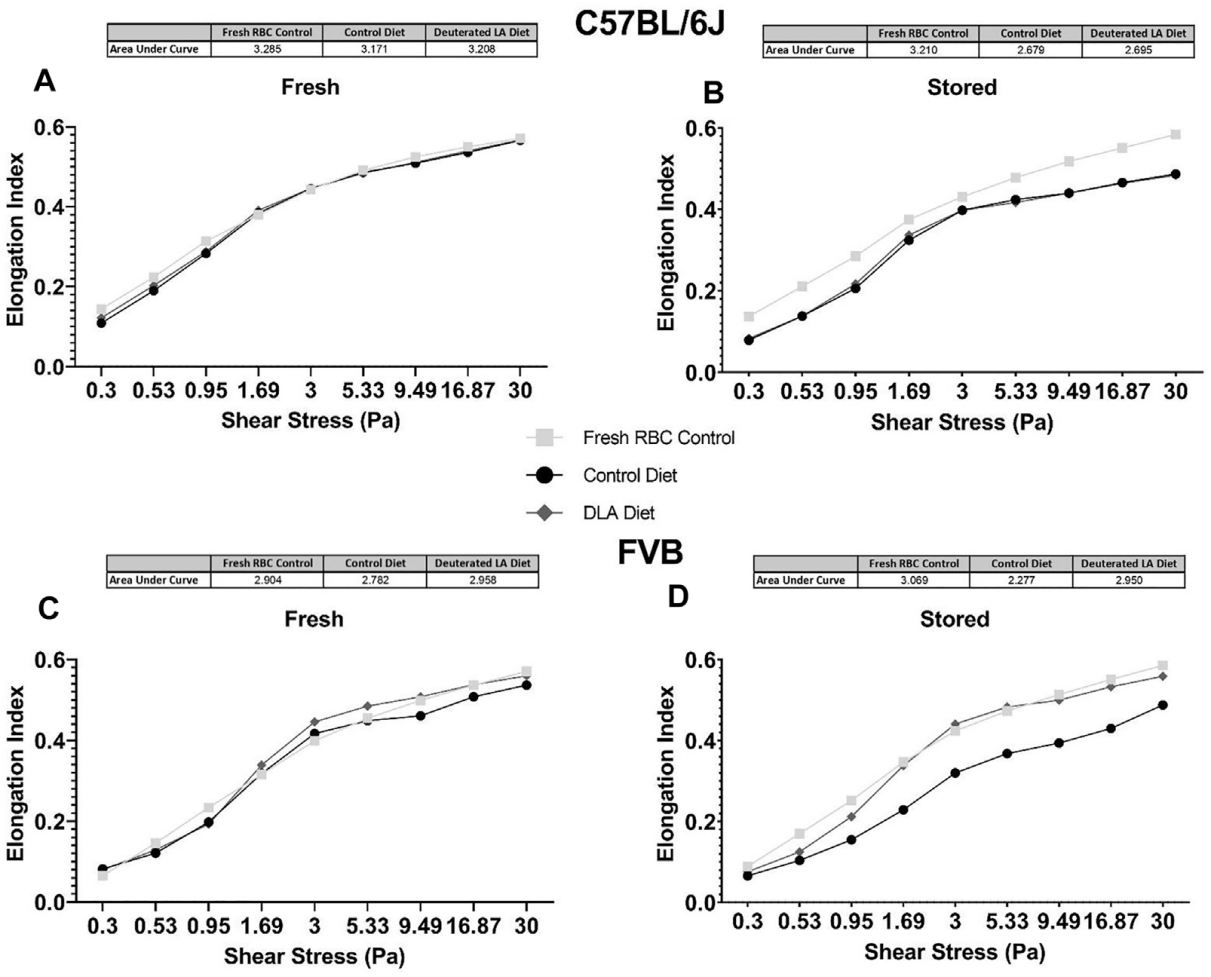
Deformability of fresh (i.e., 2 days stored) and stored RBC was assessed at various shear stresses using LORRCA. The total area under the curve was quantified for RBCs from C57BL/6 mice collected and processed the morning of the experiment (negative technical control; squares), mice consuming a control diet (circles), and mice consuming a DLA diet for 8 weeks (diamonds). Analysis was performed for C57BL/6J mice **(A, B)** and FVB mice **(C, D)**.

RBC elongation index, a measure of deformity, was measured. Increased areas under the curve suggest increased deformability, while reduced areas under the curve suggest that the RBCs are less deformable. DLA was not associated with improved deformability in fresh (Figure 6A) or stored (Figure 6B) RBCs from C57BL/6J mice. Conversely, in FVB mice, DLA was associated with improved deformability (i.e., higher elongation index) at shear stresses greater than 1.69 and 0.53 Pa after 2 and 6 days of cold storage, respectively (Figures 6C,D).

**FIGURE 7:**
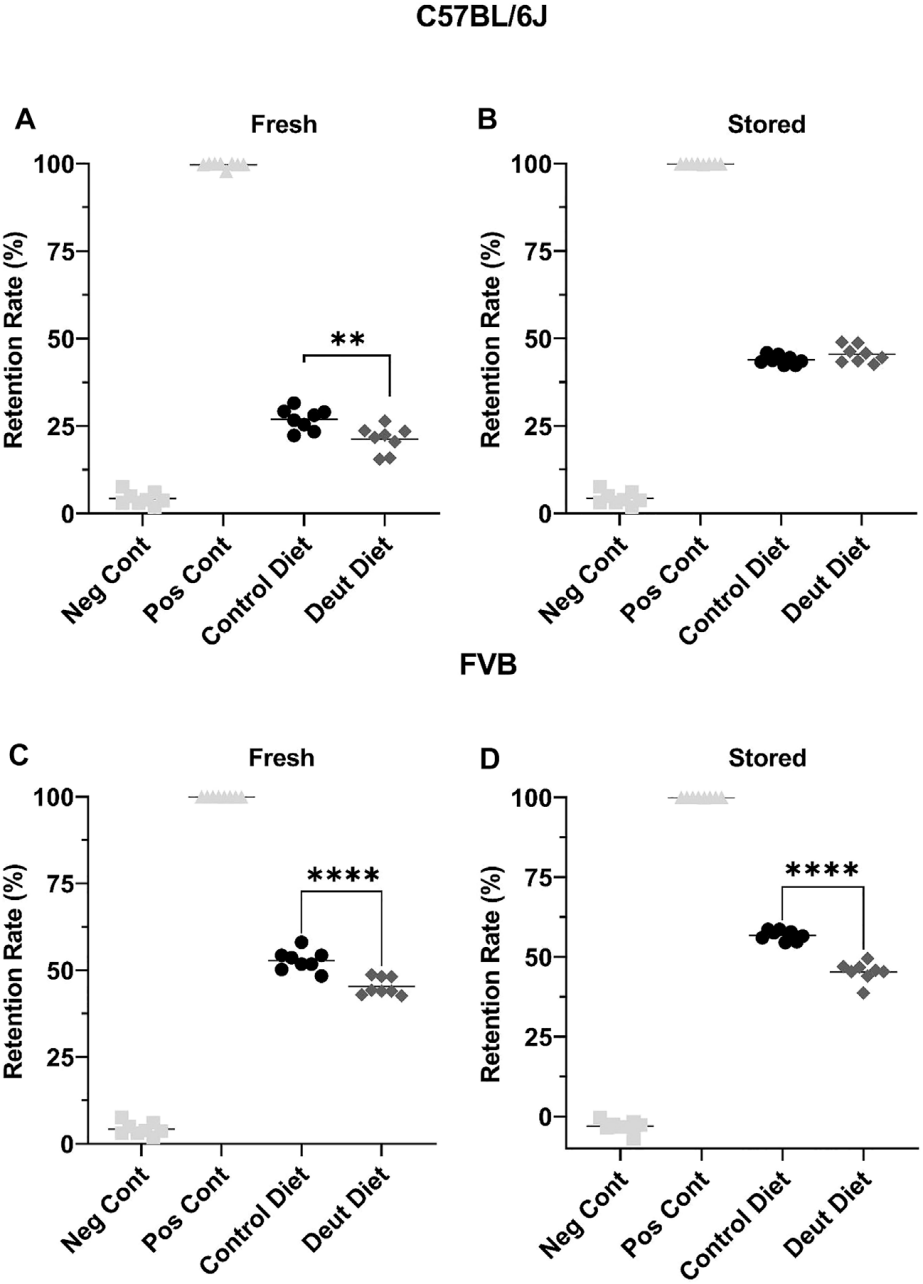
RBC deformability was assessed using microsphiltration, an *ex vivo* model of splenic sequestration. RBCs, under partial positive pressure, flow through beads of various sizes to mimic the passage through the interendothelial slits within the spleen. RBCs that are more deformable are less likely to be retained in the metal beads. Blood banks from C57BL/6J mice **(A, B)** and FVB mice **(C, D)** were introduced into the beads after 2 days of cold storage (fresh) and after 6 or 12 days of storage for the C57BL/6J and FVB mice, respectively. The retention rate was compared to RBCs from C57BL/6 mice collected the morning of the experiment (Neg Cont) and RBCs incubated with glutaraldehyde fixed RBCs (Pos Cont). Each experiment was performed twice with eight technical replicates/experiment. Group differences were analyzed by unpaired T-Test (**p < 0.01; ****p < 0.0001).

Using an *ex vivo* splenic sequestration model (i.e., microsphiltration), RBCs from FVB mice fed the DLA diet were retained at a lower rate in both “fresh” (2-day stored; 52.8 vs. 45.4%, *p* < 0.001) and stored (6-day stored) blood units (56.8 vs. 45.3%, *p* < 0.001). For C57BL/6J mice fed a DLA diet, the RBCs were retained at a lower rate in fresh, but not 12-day stored blood (Figure 7). Together, these results show that RBCs from FVB mice fed a DLA diet were more deformable at shear stresses and had reduced filterability by microsphiltration after storage, while RBCs from C57BL/6J mice fed the DLA diet did not change deformability or filterability.

### The DLA-Containing Diet was Associated With Improved 24-H PTR in FVB but Not C57BL/6J Mice

**FIGURE 8:**
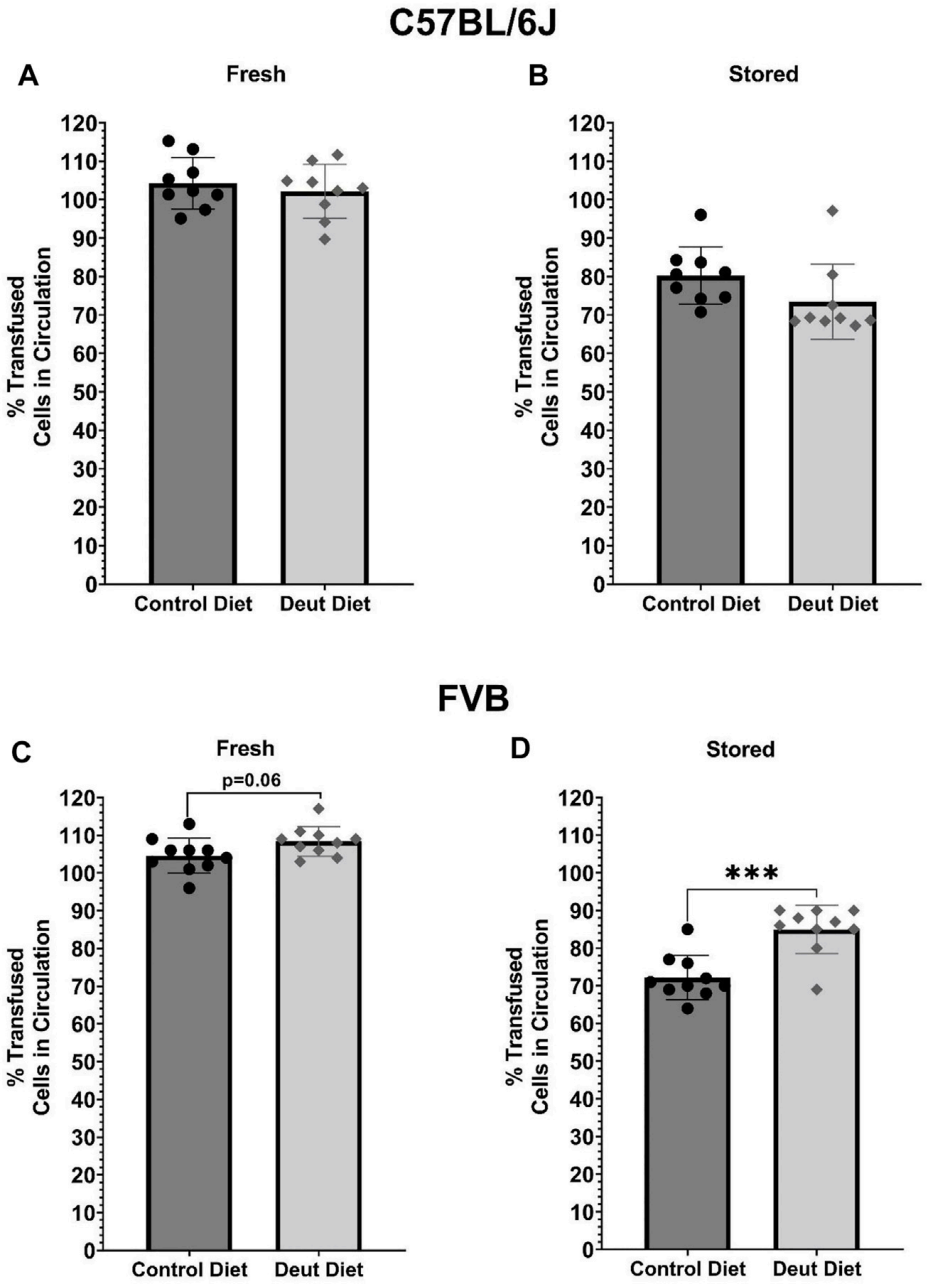
PTRs of fresh and stored RBCs. Fresh or stored biotinylated RBCs obtained from mice fed control or experimental diets were spiked 1:5 with GFP RBCs and then transfused into C57BL/6J recipient mice (*n* = 10; final volume 200 µl). Samples were obtained 5 min, 120 min, and 24 h post-transfusion. PTRs were quantified using flow cytometry as the ratio of biotinylated/GFP + RBCs relative to the biotinylated/GFP + RBC ratio in the starting blood bank. Results with C57BL/6 donor RBCs are presented in the top panels **(A, B)** and those for FVB RBCs are in the bottom panels **(C, D**; *n* = 10/group). Group differences were analyzed by unpaired T-Test (***p < 0.001).

Aliquots from fresh and stored blood units from C57BL/6J and FVB mice were transfused into C57BL/6J recipients. In C57BL/6J mice, there were no differences in 24-h PTR using either fresh (Figure 8A) or stored (Figure 8B) blood. In the model of poor RBC storage quality (i.e., FVB), DLA was associated with a borderline significant improvement in 24-h PTR using fresh blood (*p* = 0.06) and a statistically significant improved PTR after 6-day of storage (*p* < 0.001; Figure 8C). Conversely, in mice with good storage (C57BL/6J), DLA was not associated with improved PTR using fresh or stored blood (Figure 8D).

## Discussion

The results herein represent the first direct evidence linking lipid peroxidation to the RBC storage lesion. In a model of poor RBC storage quality (i.e., FVB mice), we observed a significant improvement in 24-h PTR for RBCs stored for 6 days (Figure 8). This improved PTR correlated with decreased markers of lipid peroxidation, including MDA, 4-HNE-GSH, and oxylipins derived from lipid oxidation of PUFAs (Figures 3, 4). Biomechanically, decreased oxidative damage also correlated with improved RBC deformability and filterability (Figures 6, 7).

The oxylipins measured comprised of HETEs (metabolites of AA) and HODEs (metabolites of LA). These oxylipins are generated either enzymatically via lipoxygenases or non-enzymatically through lipid peroxidation of free or esterified PUFAs. 9-HODE and 13-HODE are formed as a byproduct of the autoxidation of LA, whereas 9-HETE and 11-HETE are formed by the autoxidation of AA. Therefore, their quantification can be used as a direct measure of lipid peroxidation. In a previous study, levels of these oxylipins were shown to increase over time with the storage lesion and are dependent upon mouse genetics, especially polymorphisms in the ferrireductase STEAP3 between C57BL/6J and FVB mice (Silliman et al., 2011; Howie et al., 2019). 4-HNE is a reactive carbonyl species (RCS) formed downstream of omega-6 fatty acids including LA, AA, and DGLA. One primary mechanism for metabolizing 4-HNE is the glutathione S-transferase catalyzed conjugation of 4-HNE to GSH. As such, the level of the 4-HNE-GSH adduct is a useful marker of lipid peroxidation, a phenomenon that is exacerbated by blood storage of RBCs from donors with increased susceptibility to oxidant stress as a result of polymorphisms causing deficiency in the activity of the enzyme glucose-6-phosphate dehydrogenase (D’Alessandro et al., 2021). In C57BL/6J mice, all measured lipid peroxidation markers (i.e., MDA, 4-HNE-GSH, oxylipins) increased in both the DLA and control dietary groups after 12 days of storage, suggesting that DLA was able to partially attenuate, but not eliminate, lipid peroxidation in a mouse model with low ROS production. In line with these results, we did not observe significant differences in the PTR following transfusion of the fresh or stored C57BL/6J RBCs. In contrast, when using FVB mice, which have high ROS production and poor RBC storage quality (Zimring et al., 2014), each lipid peroxidation marker increased dramatically following RBC storage in the control diet group, but not in the DLA diet group. These results also showed that the observed decrease in oxidative damage was associated with significant improvement in PTR, compared to the dietary control group, in this mouse model of poor RBC storage quality. These results suggest a threshold level of lipid peroxidation is required for improved deformability and PTR with a further reduction of lipid peroxidation below a certain level not conferring improvement in blood quality.

*In vivo* lifespan was unchanged with DLA treatment in either rodent strain. We hypothesized that DLA reducing lipid peroxidation in the circulating RBCs with DLA would be associated with a longer RBC lifespan. In C57BL/6J mice, levels of MDA and 4-HNE were modestly decreased in non-stored RBCs with DLA treatment (Figures 3A,B) with no improvement in deformability (Figure 6A), however, filterability was improved (Figure 7A). In FVB mice, 4-HNE-GSH, as well as several oxylipins generated from the autoxidation of LA (i.e., 9-HODE, 13-HODE) were lower in RBCs from DLA treated mice, however other markers of lipid peroxidation (e.g., 9, 10-diHOME, 12, 13-diHOME, 9-HETE, and 11-HETE) were not improved with DLA treatment (Figure 4B). Some of these markers were not decreased because peroxidation is not affected by deuteration (i.e., 12, 13-diHOME), while others would be decreased by employing D6-AA, but not 13,13-D2-AA obtained from the elongation of DLA (i.e., 9-HETE, and 11-HETE). Given that FVB mice have a shorter estimated *in vivo* RBC lifespan relative to C57BL/6J mice (13% reduction; Figures 2A,B), and that DLA treatment was associated with improved deformability and filterability, it was surprising that decreasing lipid peroxidation with DLA in FVB mice did not improve RBC lifespan.

Phospholipase A2 (PLA2), or enzymes with moonlighting PLA2-like fashion such as peroxiredoxin 6 which is abundant in RBCs (Fisher, 2018), produces LPLs by hydrolyzing fatty acids at the sn2 position of phospholipids. These LPLs can then be re-acylated back to PLs or further degraded to lysophosphatidic acids. The free fatty acids (FFA) can integrate into the plasma membrane, destabilizing the lipid membrane at high concentrations (Zavodnik et al., 1997), or enzymatically metabolized to oxylipins, which have bioactive functionality. As a result, PLA2 has a significant role in fatty acid (re)cycling. Our lipidomics analyses quantified levels of FFAs and LPAs, which are a proxy for PLA2 activity. Elevated LPL and FFAs in fresh RBCs from DLA-fed mice suggest increased PLA2 activity at baseline, but not after storage. Future tracer studies will elucidate whether increased LPL levels in DLA-treated mice are due to increased basal PLA2 activity and/or decreased reacylation of LPL.

RBCs from FVB mice exhibit a pronounced storage lesion, with a 24-h PTR of 72.2% after 6 days of storage, as compared to 80.3% after 12 days of storage for C57BL/6J mice. A diet containing DLA was associated with improved deformability of stored FVB RBCs, consistent with improved PTR after 6 days of storage (85%). The significant increase in deformability was also evident in the *ex vivo* splenic microsphiltration model (Figure 7). Increased RBC deformability using LORRCA was already evident at shear stresses above 0.53 Pa, with the largest improvement measured in the 1.69–5.33 Pa range (Figure 6). In humans, shear stresses are lowest in veins (0.1–0.6 Pa), with increased shear stress in arteries (0.5–1.2 Pa), venules (∼1.5 Pa), and arterioles and capillaries (4–6 Pa) (Papaioannou and Stefanadis, 2005). Therefore, our results suggest that RBCs from mice fed DLA-containing diets would more easily traverse smaller vessels (e.g., venules, arterioles, capillaries) and the interendothelial slits of the spleen.

The improved deformability of RBCs from mice fed a DLA-containing diet may result from several potential mechanisms. For example, peroxyl radicals generated through autoxidation can propagate with adjacent membrane lipids forming a stiff cross-linked polymer mesh (Shchepinov, 2020). Additionally, RCS generated downstream of lipid peroxidation, such as 4-HNE, are highly reactive and can form adducts with membrane proteins and aminophospholipids. Thus, decreased lipid peroxidation and the resulting preservation of cytoskeletal protein structure and function could be one mechanism explaining the differences in deformability, as suggested by in silico modeling based on lipidomics data from stored RBCs (Himbert et al., 2021). Other membrane-bound proteins, such as aquaporins or band 3, whose interactome is critical to RBC cytoskeleton anchoring to the membrane (Issaian et al., 2021), may also form adducts with these RCS, preventing trans-membrane water transport and further restraining deformability (Mathai et al., 1996). Of note, polymorphisms in the gene coding for glutathione peroxidase 4 (GPX4) have been shown to correlate with increased susceptibility of stored RBCs to hemolysis following oxidant insults (Page et al., 2021). This is relevant in light of the role of GPX4, which is present and active in human RBCs in the glutathione-dependent detoxification of oxidized lipids (Stolwijk et al., 2021). Alternatively, increased autoxidation may induce vesiculation, thereby eliminating damaged cytoskeletal lipids and proteins, resulting in the transition from discocyte to spherocyte due to the progressive decrease in the surface-area-to-volume ratio (Cloos et al., 2020; Kaczmarska et al., 2020). The latter hypothesis would predict a larger population of smaller spheroid-like RBCs in the control diet relative to the DLA diet group.

In addition to biomechanical differences, PTR could also improve from biochemical changes by reducing RBC phagocytosis. The “lipid whisker model” suggests that oxidatively modified fatty acids protrude into the aqueous phase where they can be detected by macrophage-bound CD36 to enhance phagocytosis. In particular, oxidation of the fatty acid tail(s) of phospholipids was required for CD36 recognition, whereas alterations of the polar head group had no effect (Greenberg et al., 2008). When transfused, RBCs with altered fatty acid tails protruding into the aqueous environment would be more likely to be phagocytized by (e.g., splenic) macrophages expressing CD36, resulting in a lower PTR (Greenberg et al., 2008).

Sex differences in rheology and blood storage have been observed: RBCs from males are associated with reduced deformability (Szczesny-Malysiak et al., 2021), increased lipid peroxidation (Dincer et al., 2001), as well as stress-induced hemolysis after cold storage (Kanias et al., 2016; Kanias et al., 2017). These observations predict decreased PTR after cold storage in males compared to female RBCs. Indeed, male C57BL/6J and FVB mice were associated with reduced PTR relative to female mice, with improved PTR observed in FVB mice after orchiectomy (Kanias et al., 2016). Given the strain differences observed in the current study, decreasing lipid peroxidation below a certain threshold may not confer added benefit, and the DLA diet may be associated with improved deformability and PTR in male, but not female C57BL/6J mice. Future studies will be performed to test this hypothesis.

Eating a diet enriched in natural sources of antioxidants as well as high-dose antioxidation supplementation is associated with decreased production of markers of lipid peroxidation (Lubrano et al., 1989; Knight et al., 1993; Miller et al., 1998). Direct addition of antioxidants such as ascorbic acid, N-Acetylcysteine, and Trolox to stored RBCs has also been shown to be efficacious at decreasing lipid peroxidation (MDA). However, these studies did not correlate decreased lipid peroxidation with deformability or PTR (Pallotta et al., 2014; Amen et al., 2017; Antosik et al., 2018). Hypoxic storage, another mechanism for reducing the storage lesion (Nemkov et al., 2018), was associated with improved PTR, however, these studies did not directly link improved PTR with attenuated lipid peroxidation (DʼAlessandro et al., 2020).

Dietary lipids are incorporated into the RBC membrane in a dose and time-dependent matter. A relatively high dose of DLA was used in the current study (i.e., 2.1% kcal) along with a long feeding period (8 weeks) roughly corresponding to the RBC lifespan to ensure maximal incorporation of DLA into the RBC membrane. Although this diet contained 77.8% DLA, only 66.7 and 63.0% were incorporated into the RBC membranes of FVB and C57BL/6J mice, respectively. Some of the DLA was elongated and desaturated to AA with the resulting molecule protected in one of its bis-allylic positions. Future studies using lower doses and shorter feeding periods of DLA, as well as other deuterated PUFAs, alone or in combination with multiple different deuterated PUFAs (Firsov et al., 2019), will be performed to determine the minimal effective dose for decreasing lipid peroxidation and improving RBC deformability and 24-h PTR. It is possible that supplementation with deuterated PUFAs that are less abundant and more desaturated (i.e., EPA and DHA) might be equally efficacious at a lower dose, which would be more amenable to translation into human clinical studies to improve RBC quality after storage. In addition, future studies will further examine the mechanism(s) for improved deformability. Finally, as other clinical settings besides transfusion result from underlying mechanism(s) involving oxidative stress [e.g., hemolytic crises due to glucose-6-phosphate deficiency (Gerli et al., 1982) or sickle cell disease (Wu et al., 2016)], and because deuterated fatty acids involve stable isotopes and are not radioactive, they may have other applications as therapeutics in other clinical scenarios (Shchepinov, 2007; Brenna et al., 2020).

In conclusion, incorporation of DLA into RBC membranes decreases lipid peroxidation during refrigerated storage, which improves the quality of the blood product and the 24-HR PTR. These results provide a direct causal link between lipid peroxidation and the RBC storage lesion.

## Data Availability

The raw data supporting the conclusion of this article will be made available by the authors, upon reasonable request.

## Supplementary Material

Supplementary Figure S1 Body weight and food intake for C57BL/6 mice. Average C57BL/6J body weights and food intakes were monitored over the 8-week study period. For all C57BL/6J mice in each rodent cage (*n* = 5/cage; 3 cages/group) the values were recorded twice per week and the average weight/mouse was calculated based on the number of mice in each cage **(A)**. The food weight was also assessed twice per week based on the difference in the weight of the food that was added and that of the remaining food in the cage divided by the number of mice per cage **(B)**.

Supplementary Figure S2 Body weight and food intake for FVB mice. The approach used was identical to that in Supplemental Figure S2.
