# Supplementary figures and images for: Deuterated Linoleic Acid Attenuates the RBC Storage Lesion in a Mouse Model of Poor RBC Storage
Source: Front Physiol. 2022 Apr 26;13:868578. doi: 10.3389/fphys.2022.868578 (PMC9086239; doi:10.3389/fphys.2022.868578)

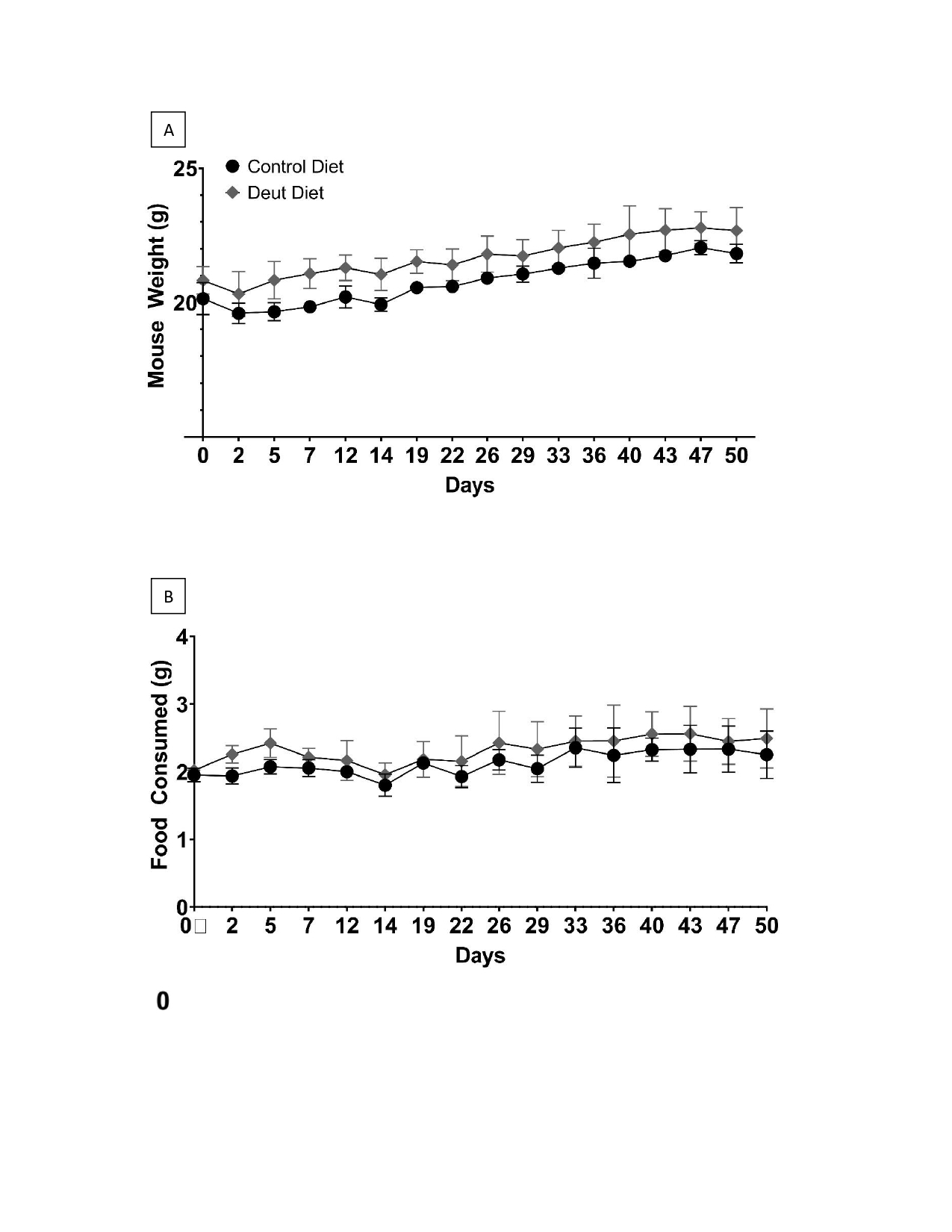

Supplement: Supplementary file 1 [file Image1.JPEG]

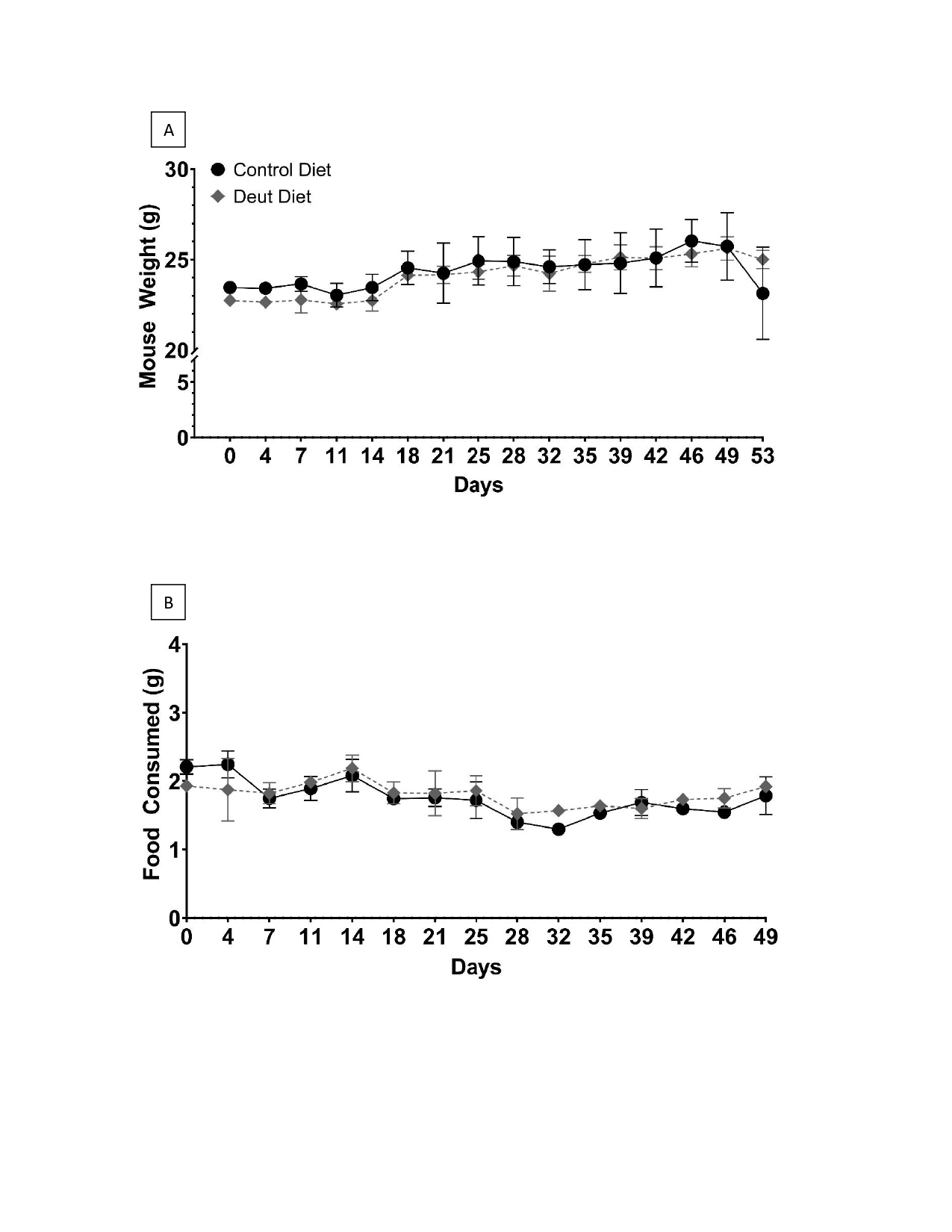

Supplement: Supplementary file 2 [file Image2.JPEG]
